# A Mendelian randomization study: Years of education and nonalcoholic fatty liver disease

**DOI:** 10.1097/MD.0000000000038761

**Published:** 2024-07-05

**Authors:** Jun Chen, Jing Li, Hongyan Qu, Ting Ning, Haoyuan Xie, Gang Lu

**Affiliations:** a Department of Acupuncture and Massage, Shaanxi University of Chinese Medicine, Xianyang, China; b Department of Chinese Medicine, The Sixth Medical Center of PLA Hospital, Beijing, China.

**Keywords:** funnel plot, leave-1-out analysis, Mendelian randomization, nonalcoholic fatty liver disease, years of education

## Abstract

Though years of education have been connected to nonalcoholic fatty liver disease (NAFLD), the exact mechanism underlying this linkage is still unknown. To investigate the causal association between years of education and NAFLD, we will use a 2-sample Mendelian randomization (MR) technique. : Genome-wide association studies data on years of education (n = 766,345) and genome-wide association studies data on nonaffiliated mental illness (n = 778,614) were screened for genetic variations as instrumental variables in the Mr-Base database. MR–Egger regression, weighted median, and inverse variance weighted were used in the MR analysis. Years of education (odds ratio = 0.63; 95% confidence interval: 0.47–0.79; *P* = 1.28 × 10^−8^) might be protective against the development of NAFLD. Among the sensitivity analyses were the following: the MR–Egger intercept test revealed *P* > .05, suggesting that there was no horizontal pleiotropy in the MR analysis and that the inverse variance weighted results were trustworthy; the Cochran *Q* test revealed *P* > .05, suggesting that there was no heterogeneity between the 2 samples; Funnel plot results demonstrated that there was no bias in the link between the measure of variability and the impact size. Leave-1-out analysis results demonstrated that no 1 single nucleotide polymorphism had a significant effect on the study’s results, showing that the MR results were stable. This study has investigated the connection between years of education and NAFLD, offering novel suggestions for NAFLD treatment and prevention.

## 1. Introduction

Nonalcoholic fatty liver disease (NAFLD) is a metabolic liver disease that is strongly linked to insulin resistance and genetic susceptibility.^[1]^ It frequently coexists with conditions associated with the metabolic syndrome, including type 2 diabetes, obesity, hypertension, and cardiovascular disorders. The hallmark of NAFLD is hepatic adiposity, which is closed to metabolic problems rather than being brought on by excessive alcohol consumption.^[2]^ Presently, NAFLD stands as one of the most common chronic liver diseases globally, with a prevalence of roughly 25.2% worldwide and up to 29.6% in Asian countries. In addition to being a liver condition, NAFLD has been linked to the onset of numerous chronic illnesses, such as diabetes, chronic kidney disease, and brain disorders.^[3]^ All of these chronic illnesses together make up the clinical features of metabolic syndrome, and NAFLD is thought to be the liver’s manifestation of metabolic syndrome. NAFLD has garnered significant attention from the international medical community and is recognized as a significant challenge in the field of public health due to the high incidence and possible health risks associated with these chronic diseases.^[4]^

Genetic background, lifestyle, dietary habits, and environmental factors are involved in the complex pathogenesis of NAFLD.^[5]^ A major contributing factor to the development of NAFLD is insulin resistance, which impairs the liver’s ability to respond to insulin. This, in turn, alters the liver’s metabolism of fat, causing hepatocytes to accumulate fat.^[6]^ Furthermore, genetic susceptibility is a major factor in the development of NAFLD, with some genetic variations having the ability to raise a person’s risk of getting the illness.^[7]^ Further study on the pathophysiology of NAFLD may lead to the development of more focused treatment approaches that will better tackle this worldwide public health concern. The possible causative relationship between years of education and a range of diseases and life expectancy outcomes has gained attention in recent decades and has seen some progress in research. For example, older adults with higher levels of education have lower susceptibility to pathological or age-related cognitive changes.^[8]^ Increased years of education have been shown to improve the frequency of sexual activity and quality of life in older males.^[9,10]^ The OECD’s Survey of Adult Skills was used by Borgonovi F and colleagues to examine the impact of human capital on health in 23 different countries worldwide.^[11]^ The study also found that educational attainment, as a measure of cognitive reserve, affects the pathological and clinical progression of Alzheimer disease. In all countries, there were significant educational differences in self-reported health, and in the majority of them, there was a strong positive correlation between years of education and self-reported health. This survey is a unique large-scale international evaluation that focuses on people aged 16 to 65.

Researchers have investigated the relationship between NAFLD and educational attainment in a cross-sectional study.^[12]^ Importantly, the study found that lower educational attainment is independently linked to a higher risk of developing NAFLD. This indicates the significance of putting in place focused intervention strategies for vulnerable populations with lower levels of education in addition to highlighting the possible influence of educational attainment on disease risk. Furthermore, another study came to the same conclusion^[13]^: health education can improve NAFLD patients’ body mass index, serum levels of triglycerides, aspartate aminotransferase, and alanine aminotransferase, as well as their gut microbiota’s diversity and their knowledge, attitudes, and compliance with regard to their health.

Mendelian randomization (MR) study, a dependable epidemiological research method, uses genetics as instrumental variables (IVs) to demonstrate causality and eliminate confounding interference, yielding stronger evidence than observational studies and second-generation randomized controlled trials in evidence-based medicine.^[14]^ More proof of the connection between years of schooling and NAFLD is provided by them. In order to investigate whether years of schooling and NAFLD are causally related, we used MR in a 2-sample design. This offered pertinent data for early prevention and treatment.

## 2. Methods

### 2.1. Data sources

This study utilized data on NAFLD and years of education from the MR-Base database, an integrated genetic epidemiology resource that lets users investigate the connections between different genetic variations and health outcomes. The ID ieu-a-1239 specifically identified the data for years of education, while the ID ebi-a-GCST90091033 specifically identified the data for NAFLD. These data were chosen to ensure broad representativeness and high reliability of the study based on their high-quality meta-analysis results and contributions from numerous global centers. Table 1 provides a comprehensive explanation of the facts.

**Table 1:** Description of the genome-wide association study data sets.

Phenotype	Instrumental variable	Ancestry	Sample size	Source	First author, yr
Exposures					
Years of education	317	European	766,345	MR-Base	Lee, 2018
Outcomes					
NAFLD	304	European	778,614	MR-Base	Ghodsian N, 2021

MR = Mendelian randomization, NAFLD = nonalcoholic fatty liver disease.

MR studies do not involve direct intervention or manipulation of research subjects. Therefore, the study does not require ethical review.

### 2.2. Selection of IVs

An essential component of MR analysis is choosing the right IVs. Single nucleotide polymorphisms (SNPs) from genome-wide association studies (GWAS) were carefully chosen for this investigation. To act as IVs, a total of 317 independent SNPs with *P* values less than 5 × 10^−8^ were selected based on their high correlation with educational attainment from GWAS data connected to years of education. From the relevant GWAS, 304 independent SNPs were chosen as IVs for NAFLD. To confirm their independence, all of the chosen SNPs underwent extensive linkage disequilibrium (LD) testing. The domain width was set to 10,000 kb, and the LD coefficient *R*^2^ was set to 0.001. If a specific exposure SNP was missing, we used LD markers to select proxy SNPs, with the minimum *R*^2^ value for LD set to 0.8 and the minimum minor allele frequency threshold set to 0.3, to ensure the representativeness and effectiveness of the proxy SNPs.

### 2.3. Study design

**Figure 1.**
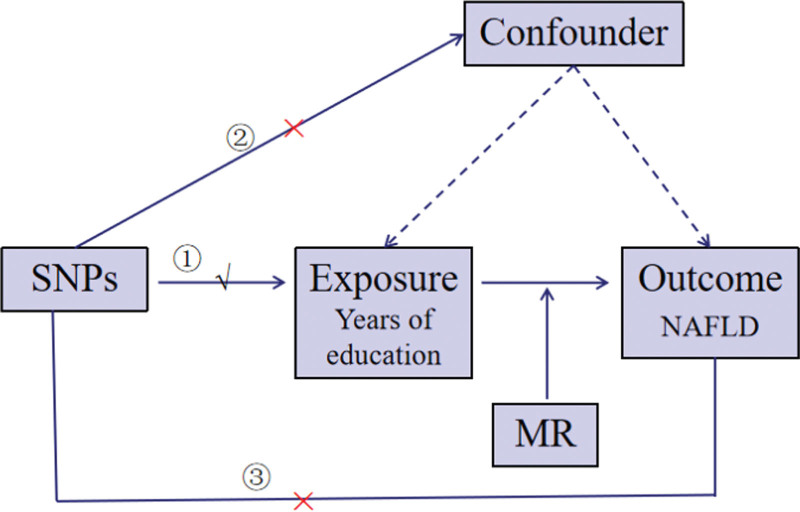
The Mendelian randomization study design.

The 2-sample MR method was used in this study, allowing genetic IVs from 1 sample and outcome data from another to be used. NAFLD is regarded as the outcome variable, and years of schooling as the exposure variable. Using MR analysis, the possible causal link between the 2 was investigated. For MR analysis to be valid, 3 fundamental assumptions must be met: The assumption of relevance: the IV has a strong and distinct relationship with the exposure variable; The assumption of independence: the IV is unrelated to any confounding variables; The assumption of exclusivity: the exposure variable is the only way that the IV influences the result. Verification of these assumptions is a crucial step in the analysis, which helps to ensure the accuracy of causal inferences. The study design is depicted in Figure 1.

### 2.4. Statistical analysis

**Figure 2.**
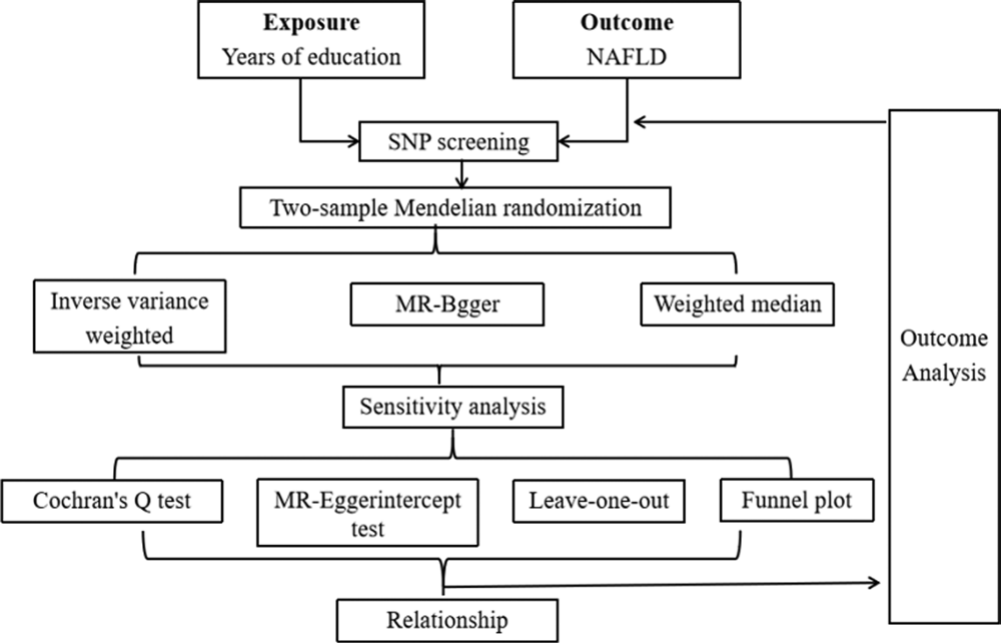
The flow chart.

R language (version 4.3.1), and the “Two-Sample MR” package (version 0.5.7) were used in this study’s MR and sensitivity analyses. Inverse variance, weighted, MR–Egger, and weighted median (WM) MR analysis was used to examine if years of education and NAFLD were causally related. To determine if years of education and NAFLD were causally related, MR analyses employed inverse variance, weighted, MR–Egger, and WM. Sensitivity analysis evaluates the stability and dependability of the findings from MR analysis.^[6]^ As seen in Figure 2, we evaluated the MR results using the Cochran *Q* test, MR–Egger intercept test, leave-1-out analysis, and Funnel plot.

## 3. Results

### 3.1. MR analysis

In this study, MR study was performed using multiple approaches to investigate potential causal correlations between years of schooling and the risk of NAFLD.

**Figure 3.**
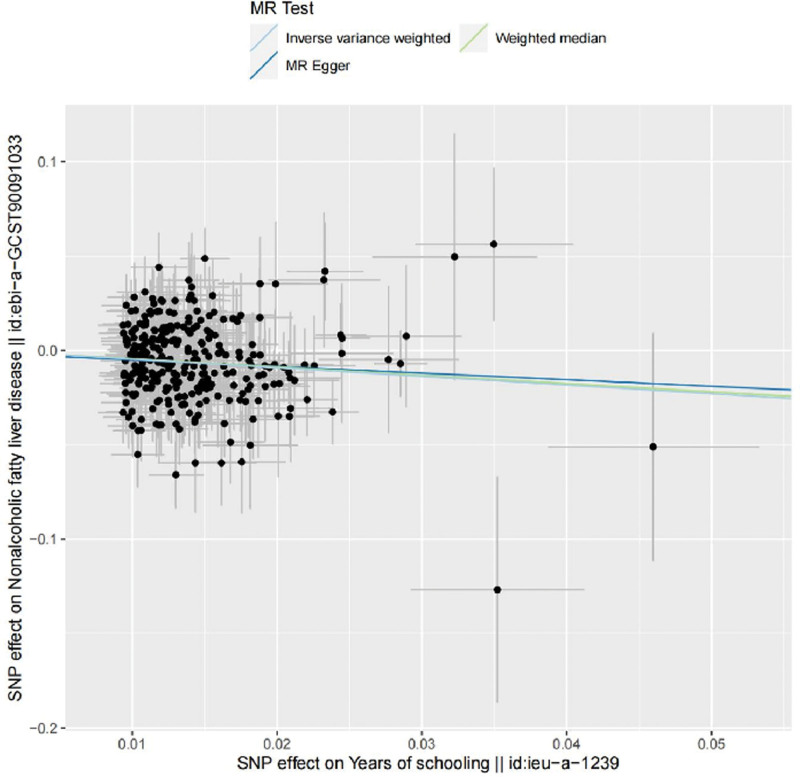
The Scatter plot of years of education and NAFLD. NAFLD = nonalcoholic fatty liver disease.

An increase in years of education was found to considerably reduce the chance of developing NAFLD, according to analysis utilizing inverse variance weighted (IVW) approach. In particular, the odds ratio (OR) was 0.63, achieving a statistically significant level with a *P* value <0.05 and a 95% confidence interval (CI) spanning from 0.47 to 0.79. This finding provides significant support for the idea that years of education operate as a protective factor against NAFLD, indicating that an individual’s chance of getting NAFLD may drop by almost 37% for every extra year of education. The data analysis was also conducted using the MR–Egger method. Notably, the direction of the effect estimate was consistent with that of the IVW method, suggesting that an increase in years of education is associated with a decreased risk of NAFLD. This is despite the fact that the OR obtained from this method was 0.70 (95% CI: 0.08–1.32), with a *P* value >0.05 and thus not achieving statistical significance. This consistency suggests that, even with the MR–Egger method’s marginally reduced statistical power in this investigation, its directional result is consistent with the IVW finding, supporting the notion that years of schooling serve as a protective factor against NAFLD. The preceding conclusions were further confirmed by the WM analysis. With a *P* value <0.05, the WM method’s OR of 0.64 (95% CI: 0.41–0.87) and a negative connection between years of education and the risk of NAFLD were also confirmed. The MR–Egger method’s direction of effect was similar to that of the IVW and WM methods, both of which showed strong negative correlations when the results from the 3 MR techniques previously reviewed were combined. However, its *P* value did not reach a significant level. Thus, we have reason to believe that the results of the IVW technique, suggesting that years of schooling could be a protective factor against the development of NAFLD, are supported. The key findings of the MR analysis are shown in Table 2. Figure 3 presents a scatter plot graphically.

**Table 2:**
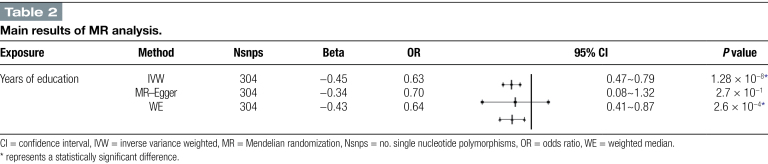
Main results of MR analysis.

### 3.2. Sensitivity analysis

To ensure the robustness and validity of the MR analysis results, we conducted a multifaceted sensitivity analysis.

#### 3.2.1. Cochran *Q* test for heterogeneity initially

To investigate the heterogeneity between the IVs, we used Cochran *Q* test. A *P* value larger than 0.05 in the results suggested that there was no significant heterogeneity among the IVs that were included. This suggested that the influence of each genetic marker on the years of education was relatively consistent, which was consistent with the requirement for MR analysis – that was, the genetic markers that were chosen should be legitimate causal mediators between years of education and NAFLD. The results are displayed in Table 3.

**Table 3:** Main results of the MR sensitivity analysis.

Exposure	Cochran *Q* test			MR–Egger intercept test		
	Method	Cochran *Q*	*P*	Egger intercept	SE	*P*
Years of education	MR–Egger	319.3	.23*	−0.0015	0.0043	.72*
	IVW	319.4	.24*			

IVW = inverse variance weighted, MR = Mendelian randomization, SE = standard error.

* represents a statistically significant difference.

#### 3.2.2. MR–Egger intercept test for horizontal pleiotropy

The MR–Egger Intercept test was employed to assess horizontal pleiotropy. The findings revealed a *P* value larger than 0.05, which suggested that the current investigation did not contain any meaningful evidence of horizontal pleiotropy. This implied that the chosen genetic markers were not concurrently impacting the incidence of NAFLD via unidentified mechanisms, thus confirming the validity of the IVW technique and the reliability of the findings. These results are shown in Table 3 and are represented graphically in Figure 3 by the Egger regression line.

#### 3.2.3. Leave-1-out analysis for robustness

**Figure 4.**
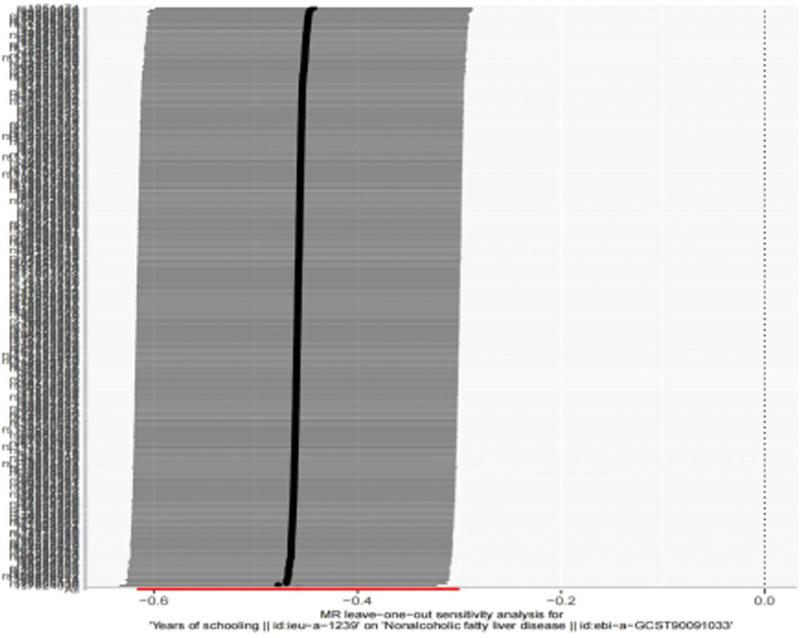
The sensitivity analysis results of “Leave-one-out” between years of education and NAFLD. NAFLD = nonalcoholic fatty liver disease.

We performed a leave-1-out analysis to evaluate the effect of individual genetic markers on the overall MR results. The findings showed that the influence of each included SNP on the overall effect size was negligible, with the effect sizes remaining extremely close to the total effect size, when each instrumental variable was sequentially removed and the analysis was re-performed. This suggested that the overall research conclusion was stable and is not significantly affected by outliers in individual genetic markers, even if a single SNP was excluded. Figure 4 provides a clear illustration of how to evaluate this stability.

#### 3.2.4. Funnel plot for publication bias

**Figure 5.**
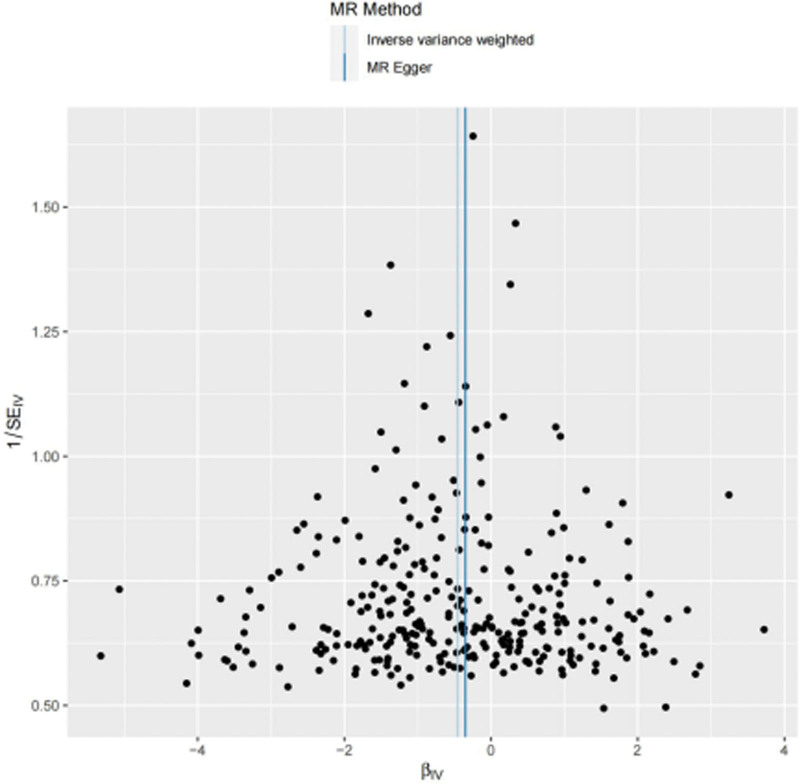
Funnel plot test of years of education and NAFLD. NAFLD = nonalcoholic fatty liver disease.

Lastly, in an effort to identify any biases, we used a Funnel plot to investigate the relationship between the measures of variability and the size of the effect sizes. The findings demonstrated that the effect estimate points were dispersed equally throughout the plot and displayed an ordinary inverted funnel shape devoid of any discernible asymmetry, suggesting that the study does not contain any substantial publication bias or other types of systematic bias. This result increases our trust in the validity of the conclusions drawn from the MR analysis, as shown in Figure 5.

## 4. Discussion

The MR-Base database is a large genetic resource that we fully utilized in the MR study investigating the association between years of education and the risk of NAFLD. The database compiles a plethora of worldwide genetic data, and its sizable sample size gives our analysis strong statistical power. This makes it possible for us to determine possible causal relationships more precisely.^[15]^

Nevertheless, the study also recognizes that the geographic and ethnic representativeness of the available data is essentially what limits it. In particular, all data used in this study originate from the European population, which somewhat restricts the applicability of the findings of the study. The following points provide an in-depth discussion of this limitation:

1. Ethnic-Specific Genetic Variations and Environmental Interactions. Genetic variation patterns may differ amongst ethnic groups,^[16]^ and these differences may have a particular correlation with the number of years of education and the risk of NAFLD. Additionally, people from various ethnic backgrounds could have varied lifestyles, nutritional preferences, socioeconomic contexts, and access to healthcare. The development and course of NAFLD may be influenced by the interaction of these environmental variables and genetic predispositions. Hence, research results derived only from European populations might not be readily generalized to other ethnic groups, particularly those with notable genetic and environmental distinctions.
2. Extrapolation of Genetic Effects. The protective effect of years of education on the risk of NAFLD, as derived from the European population in this study, may differ among other ethnic groups due to the likelihood that the size and direction of genetic influences may vary among different ethnicities.^[17]^ For example, some genetic markers linked to years of education might not be present in other ethnic groups, or their correlation with NAFLD might be different in strength. Studies of cross-ethnic validation are required to solve this problem of extrapolating genetic effects.
3. Environmental Adaptability and Cultural Differences. Years of education can have varying effects on a person’s health-related behaviors, knowledge acquisition, and use of medical services depending on the sociocultural and geographic setting.^[18,19]^ Cultural factors may impact how the number of years of education precisely displays its preventative effects against NAFLD. Examples of these factors include the quality of the education system, the health-related content of education, and social opinions on the importance of education. As a result, it is possible that some conclusions about the European educational system and its association with the risk of NAFLD won’t apply to other geographical areas.

The results of this investigation have significant benefits. First of all, it offers more trustworthy and precise data to back up the association between educational attainment and NAFLD. It might make it easier for legislators and healthcare experts to create effective preventative and management plans. Second, the study’s conclusions underscore the significance of the formative years. Numerous studies have demonstrated a correlation between years of formal education and improved health and well-being.^[20]^ Education can have a significant impact on health in addition to imparting knowledge and skills. People’s lifestyles have changed significantly as the economy grows and living conditions rise, and this has led to a steady rise in the incidence of NAFLD.^[21]^ It suggests that a healthy lifestyle, which includes a balanced diet and proper exercise, may be more important to people with higher education levels, and that this could lower the chance of developing NAFLD. Thirdly, a person’s degree of understanding and awareness of NAFLD may also be influenced by their years of education. The prognosis for NAFLD can be improved with more knowledge about the condition and how to prevent and treat it. Therefore, in order to enhance general health, society, and the government should prioritize funding and enhancing education. In conclusion, the results also advance our knowledge of NAFLD. NAFLD is a condition linked to contemporary dietary patterns and lifestyle choices.

## 5. Conclusions

The present study identified the relationship between years of education and NAFLD. We can better understand the pathogenesis of NAFLD and provide more targeted measures for prevention and treatment. In conclusion, the results of this study have important implications for revealing the relationship between years of education and NAFLD, providing new perspectives and strategies for the prevention and management of NAFLD.

## Author contributions

**Conceptualization:** Jun Chen, Gang Lu.

**Data curation:** Jun Chen.

**Formal analysis:** Ting Ning.

**Funding acquisition:** Jun Chen.

**Investigation:** Jun Chen, Jing Li.

**Methodology:** Jun Chen, Jing Li, Haoyuan Xie.

**Project administration:** Hongyan Qu, Ting Ning.

**Resources:** Jing Li, Hongyan Qu, Haoyuan Xie.

**Software:** Hongyan Qu, Haoyuan Xie.

**Supervision:** Hongyan Qu.

**Validation:** Hongyan Qu, Ting Ning, Haoyuan Xie, Gang Lu.

**Visualization:** Ting Ning, Gang Lu.

**Writing – original draft:** Gang Lu.

**Writing – review & editing:** Gang Lu.
